# Association of Transcription Factor Gene *LMX1B* with Autism

**DOI:** 10.1371/journal.pone.0023738

**Published:** 2011-08-25

**Authors:** Ismail Thanseem, Kazuhiko Nakamura, Ayyappan Anitha, Shiro Suda, Kazuo Yamada, Yoshimi Iwayama, Tomoko Toyota, Masatsugu Tsujii, Yasuhide Iwata, Katsuaki Suzuki, Hideo Matsuzaki, Keiko Iwata, Toshiro Sugiyama, Takeo Yoshikawa, Norio Mori

**Affiliations:** 1 Department of Psychiatry and Neurology, Hamamatsu University School of Medicine, Hamamatsu, Japan; 2 Research Center for Child Mental Development, Hamamatsu University School of Medicine, Hamamatsu, Japan; 3 Department of Psychiatry, Jichi Medical University, Shimotsuke, Japan; 4 Laboratory of Molecular Psychiatry, RIKEN Brain Science Institute, Saitama, Japan; 5 Faculty of Sociology, Chukyo University, Toyota, Aichi, Japan; 6 Aichi Children's Health and Medical Center, Obu, Aichi, Japan; 7 Department of Child and Adolescent Psychiatry, Hamamatsu University School of Medicine, Hamamatsu, Japan; Chiba University Center for Forensic Mental Health, Japan

## Abstract

Multiple lines of evidence suggest a serotoninergic dysfunction in autism. The role of *LMX1B* in the development and maintenance of serotoninergic neurons is well known. In order to examine the role, if any, of *LMX1B* with autism pathophysiology, a trio-based SNP association study using 252 family samples from the AGRE was performed. Using pair-wise tagging method, 24 SNPs were selected from the HapMap data, based on their location and minor allele frequency. Two SNPs (rs10732392 and rs12336217) showed moderate association with autism with p values 0.018 and 0.022 respectively in transmission disequilibrium test. The haplotype AGCGTG also showed significant association (p = 0.008). Further, *LMX1B* mRNA expressions were studied in the postmortem brain tissues of autism subjects and healthy controls samples. *LMX1B* transcripts was found to be significantly lower in the anterior cingulate gyrus region of autism patients compared with controls (p = 0.049). Our study suggests a possible role of *LMX1B* in the pathophysiology of autism. Based on previous reports, it is likely to be mediated through a seretoninergic mechanism. This is the first report on the association of *LMX1B* with autism, though it should be viewed with some caution considering the modest associations we report.

## Introduction

Autism and other developmental disabilities, clinically referred to as autism spectrum disorders (ASDs), are characterized by impairments in communication skills and social interaction, and the presence of repetitive stereotyped behaviors and interests. It is typically diagnosed by the age of three and has a prevalence rate of 60-70 per 10,000 children in broader diagnostic criteria as per the most recent estimates [1]. ASDs are considered to be among the most heritable of all psychiatric disorders. A recent largest population based twin study comprised of 10,895 twin pairs, reported 80% heritability for ASDs [2], confirming the previously reported heritability estimates [3], [4]. Linkage, candidate gene and whole genome association studies have suggested several genes and chromosomal regions associated with the disorder. However, none of these known causes individually account for more than 1–2% of the cases, and specific genetic mechanisms underlying the heritability of the disorder still remain largely cryptic. It was found that many different genetic changes in unrelated genes can cause indistinguishable ASD features; this genetic heterogeneity necessitate the need to look for more potential candidate genes associated with the disorder.

The LIM homeodomain transcription factor 1b (LMX1B) was initially characterized as a key regulator of the normal dorsoventral patterning in the developing limbs [5]. Several mutations reported in this gene have been found to lead to the pleiotropic phenotype, the nail platella syndrome [6]–[8]. Later, the role of *Lmx1b* in the development and maintenance of serotoninergic (5HTergic) neurons in the central nervous system (CNS) was reported, and thereafter, underlying mechanisms were studied in detail. *Lmx1b* knock-out mice were found to be lacking the entire central 5HTergic neurons [9], [10]. Further, it was shown that overexpression of *Lmx1b* enhances differentiation of mouse embryonic stem cells into 5HT neurons [11]. In addition to its role in the development of central 5HTeregic neurons, *Lmx1b* is also required for the normal biosynthesis of 5HT in adult brain, and possibly for the regulation of normal functions of 5HTergic neurons [12].

A role of 5HTergic system in the pathophysiology of autism was proposed based on following observations, a) hyperserotonemia in the whole blood cells and platelets of 25–50% of patients with autism [13], [14], b) depletion of tryptophan, the 5HT precursor, in ASD patients increased some stereotype behaviors associated with the disorder [15], c) treatment with selective serotonin reuptake inhibitors has shown to be effective in ameliorating the repetitive and/or compulsive behaviors in some autistic individuals [16] and d) recent neuroimaging studies have shown low levels of brain 5HT synthesis in autistic children [17] and reduction in serotonin transporter (SLC6A4) binding in different brain regions of both children and adults with the disorder [18], [19]. Compliant with these reports, several genetic association studies involving genes in the 5HT metabolism with a focus on the *SLC6A4* were also attempted. While several *SLC6A4* polymorphisms were shown to be associated with the disorder in some studies [20], [21], others failed to replicate the findings [22].

Taking together, these results provide compelling, though inconsistent evidence for the role of 5HTergic system in the pathophysiologic mechanism of ASDs. In view of the importance of LMX1B in the development of 5-HTergic neurons, it would be interesting to study its role in autism. Here we performed a trio-based study to examine the association of *LMX1B* with autism. We also assessed any alterations in the expression *LMX1B* in the postmortem brain samples of autism patients as compared to healthy controls.

## Results

### Single SNP TDT

Mendelian inheritance inconsistencies were not observed for any of the SNPs. For each SNP, >99% of the genotypes were scored; none of the SNPs showed deviation from HWE.

The results of TDT analysis are shown in Table 1. rs10732392 (p = 0.018; OR = 1.764; 95% CI for OR 1.095–2.842) and rs12336217 (p = 0.022; OR = 1.748; 95% CI for OR 1.076–2.841) showed significant associations with autism. However, these associations did not withstand the multiple testing correction. Overtransmission was observed for the minor allele A (62.82%) of rs10732392 and for minor allele G (62.67%) of rs12336217.

**Table 1:** Single SNP TDT results of *LMX1B* SNPs in 252 trio samples.

Marker	db SNP ID	Genomic	Variation*	Location	Minor allele	T (%)‡	*p*-value^§^
		Location			frequency†		
SNP 1	rs10732392	129396037	G:A	Intron 2	0.078	48.92	***0.018***
SNP 2	rs10760444	129396434	A:G	Intron 2	0.449	48.23	0.214
SNP 3	rs10448285	129397014	C:T	Intron 2	0.376	50.64	0.601
SNP 4	rs12336217	129399870	A:G	Intron 2	0.075	48.98	***0.022***
SNP 5	rs7858338	129406644	T:C	Intron 2	0.26	51.61	0.085
SNP 6	rs11793373	129407543	G:A	Intron 2	0.252	50.6	0.513
SNP 7	rs10819190	129408513	G:A	Intron 2	0.414	49.56	0.739
SNP 8	rs6478750	129409198	T:C	Intron 2	0.408	49.91	0.948
SNP 9	rs12555734	129411242	C:A	Intron 2	0.24	51.25	0.16
SNP 10	rs13285227	129413298	C:T	Intron 2	0.348	49.11	0.439
SNP 11	rs944103	129413490	G:A	Intron 2	0.472	49.05	0.526
SNP 12	rs12555176	129414303	G:T	Intron 2	0.074	50.11	0.809
SNP 13	rs7854658	129414938	G:A	Intron 2	0.21	50.57	0.486
SNP 14	rs10987386	129416317	C:T	Intron 2	0.191	49.5	0.519
SNP 15	rs12551234	129417809	G:C	Intron 2	0.407	49.92	0.949
SNP 16	rs7853174	129419990	G:A	Intron 2	0.394	49.04	0.452
SNP 17	rs10819194	129422023	G:A	Intron 2	0.422	51.78	0.189
SNP 18	rs4322101	129428677	A:G	Intron 2	0.416	51.19	0.37
SNP 19	rs7030919	129438872	A:G	Intron 2	0.115	49.49	0.37
SNP 20	rs3737048	129458092	G:T	Intron 6	0.107	50.39	0.474
SNP 21	rs10987413	129459438	G:A	3′	0.333	50.65	0.56
SNP 22	rs10760450	129459628	C:T	3′	0.21	50.58	0.475
SNP 23	rs10733682	129460914	G:A	3′	0.486	51.27	0.41
SNP 24	rs4083644	129461714	C:T	3′	0.28	49.93	0.943

T: Transmitted.

*Common allele is listed first.

†Based on the parental genotypes of 252 trios.

‡T% of common allele is listed, § Computed on the basis of likelihood ratio test; significant p-values (<0.05) are indicated in bold italics.

### LD analysis

**Figure 1:**
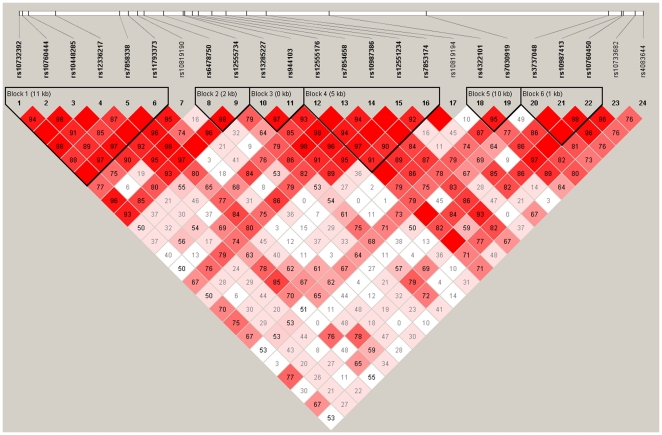
Haploblock structure of *LMX1B.* Six haplotype blocks were identified based on D' values calculated from 252 trios.

LD analysis based on D' values identified six distinct haploblocks across *LMX1B* gene. The first block consists of SNPs 01 to 06, the second block SNPs 08 and 09, the third block 10 and 11, fourth block 12 to 16, fifth block 18 and 19 and the sixth block included SNPs 20 to 22 (Figure 1).

### Haplotype TDT

The results of haplotype TDT is given in Table 2. Based on the LD structure of *LMX1B,* associations of haplotypes in the six haploblocks were analysed. The haplotype AGCGTG of the first block showed significant association with autism (p = 0.008).

**Table 2:** Haplotype associations of SNPs belonging to the six LD blocks of *LMX1B*, in 252 trios.

Block	Haplotype*	Frequency	T(%)	Individual *p-*	Permutation *p*-	Block *p*-
				value†	value‡	value
Block 1 (SNPs 01–06)	GGTATG	0.355	51.67	0.6291	1	
	GACATA	0.25	48.81	0.7487	1	
	GACACG	0.244	45.71	0.2568	0.994	
	AGCGTG	0.073	66.13	***0.0079***	0.114	
	GACATG	0.052	51.42	0.8461	1	
	GGTACG	0.014	30.77	0.1658	0.97	0.096
Block 2 (SNPs 08–09)	CC	0.406	50.23	0.9432	1	
	TC	0.353	54.03	0.2242	0.987	
	TA	0.239	44.23	0.1255	0.892	0.258
Block 3 (SNPs 10–11)	CG	0.525	48.4	0.6123	1	
	TA	0.345	52.71	0.4094	1	
	CA	0.126	48.79	0.8046	1	0.731
Block 4 (SNPs 12–16)	GGCGA	0.379	53.41	0.3114	0.998	
	GGCGG	0.209	45.31	0.2362	0.991	
	GACCG	0.201	48.99	0.8072	1	
	GGTCG	0.119	55.41	0.2624	0.994	
	TGTCG	0.071	48.81	0.8455	1	0.595
Block 5 (SNPs 18–19)	AA	0.58	52.42	0.4476	1	
	GA	0.304	47.25	0.1587	0.966	
	GG	0.112	53.61	0.4772	1	0.354
Block 6 (SNPs 20–22)	GGC	0.35	55.39	0.111	0.868	
	GAC	0.332	48.19	0.59	1	
	GGT	0.21	47.63	0.5365	1	
	TGC	0.107	46.45	0.4947	1	0.512

T: Transmitted / (Transmitted + Untransmitted).

‡10,000 permutations.

*All possible combinations of haplotypes with frequency >0.01 †Significant *p*-values (<0.05) are indicated in bold italics.

### *LMX1B* expression in the postmortem brains

**Figure 2:**
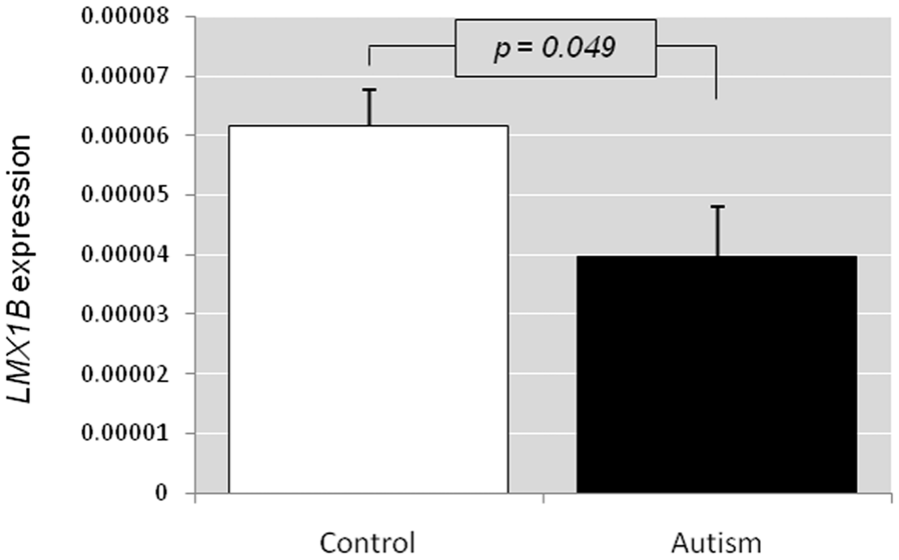
*LMX1B* expression in the brain. *LMX1B* expression in the anterior cingulate gyrus region of the brain of autism patients compared to that of control samples.

No significant difference in age, sex and postmortem intervals was observed between autism and control groups in all the brain regions (ACG, MC and THL). There was a significant difference in *LMX1B* expression between the autism and control group in the ACG (p = 0.049) (Figure 2). Expression was significantly lower in autism groups with a fold change of (2^−△△C^T) 0.43. No *LMX1B* expression could be detected in the other two brain regions (MC and TH).

## Discussion

In this study, we examined the association of the transcription factor gene *LMX1B* with autism in Caucasian population. In the trio-based study, we found nominal associations for two SNPs (rs10732392 and rs12336217) and a haplotype with autism. To the best of our knowledge, this is the first study which reported an association between *LMX1B* and autism; a previous study reported the association between *LMX1B* and schizophrenia [23], which is also a neurodevelopmental disorder. Both the SNPs which are found to be associated with the disorder are located in the introns (intron 2) and may lack any direct functional importance. We also found that the *LMX1B* mRNA expression in general, is rather low in adult brain; detected only in ACG. However, *LMX1B* mRNAs were found to be significantly lower in the ACG of autistic brains than the similar regions of control brain tissues.

Multiple lines of evidence suggested a serotoninergic dysfunction in many patients with autism, although the results are still inconclusive. Involvement of several transcription factors are reported in the 5HTergic differentiation. In mammalian CNS, a sequential activation of transcription factors in the hindbrain, starting with the regulation of the expression of *Nkx2-2* by the *Shh* signaling pathway, has been proposed [9]. It was observed that 5HT neurons are absent in the mice lacking *Nkx2-2* [24]. It occupies the highest hierarchical position in the genetic cascade that involved in the development of 5HT neurons. Another transcription factor *Pet1*, expressed in the post mitotic 5HT neurons was reported to be the terminal differentiation factor, which acts in the final step of the transcriptional cascade that establishes the final identity of 5HT neurons. Mice lacking *Pet1* had 70–80% fewer 5-HT neurons than normal mice. The *Lmx1b* ablation does not affect the expression Nkx2.2 and Shh [9], [25] putting these factors upstream of Lmx1b. However, during development, *Lmx1b* precedes *pet1*, and *Lmx1b* knock-out mice showed loss of *Pet1* expression [10]. *In vivo*, *Pet1* expression was increased in neurons overexpressing *Lmx1b* [11]. Thus, *Lmx1b* has been proposed as an essential link between Nkx2.2 and Pet1 in the genetic cascade that controls the early specification and terminal differentiation of 5HTergic neurons in the hindbrain. Lmx1b expression was shown to be the rate limiting step in this cascade of events for specifying the 5HT phenotype [11]. Further, Lmx1b, together with Pet1, is also involved in the serotonin metabolism as it controls a set of molecules essential for the serotonin synthesis (TPH2), vesicular transport (VMAT2) and reuptake after synaptic release (SLC6A4) in the developing as well as adult brain [10], [12].

ACG region plays important role in the pathophysiology of autism as shown by previous reports [26], [27]. Our positron emission tomography studies had shown that a reduction in SLC6A4 binding in the cingulate cortices is associated with an impairment of social cognition in autistic subjects [19]. The present finding of reduced *LMX1B* expression in the ACG of autism group, therefore, could have some deleterious effects on the serotonergic system, given the role of LMX1B in the differentiation of 5HT neurons in developing brain, and in the maintenance of 5HT system in adult brain.

In conclusion, we report a possible association of the transcription factor *LMX1B* with autism pathogenesis. However, our results should be interpreted with some caution, given the limitations in sample size of postmortem brain samples and the modest associations we found in genetic and gene expression studies.

## Materials and Methods

### Subjects

DNA samples from trio families recruited to the Autism Genetic Resource Exchange [28] were used for the single nucleotide polymorphism (SNP) association study. We selected 252 trios families with male offspring scored for autism. Only Caucasians (white) were selected and non-idiopathic autism cases were excluded.

### Brain samples

Frozen postmortem brain tissues from autistic patients and controls were provided by the Autism Tissue Program (ATP; Princeton, NJ; http://www.autismtissueprogram.org) and Harvard Brain Tissue Research Center (HBTRC; Belmont, MA; http://www.brainbank.mclean.org/). Tissues were obtained from three brain regions important in cognitive and behavior processing namely a) anterior cingulate gyrus (ACG- 8 autism and 13 controls), b) motor cortex (MC- 7 autism and 8 controls), and c) thalamus (THL-8 autism and 9 controls). The demographic features of the samples are described in Table 3.

**Table 3:** Postmortem brain tissue information.

Sample ID^a^	Diagnosis	Age (years)	Gender	PMI (hours)	Race	Cause of death	Brain regions^b^
UMB 818	Control	27	M	10	Caucasian	Multiple injuries	ACG
UMB 1065	Control	15	M	12	Caucasian	Multiple injuries	ACG, THL
UMB 1297	Control	15	M	16	African American	Multiple injuries	ACG, MC, THL
UMB 1407	Control	9	F	20	African American	Asthma	ACG, MC, THL
UMB 1541	Control	20	F	19	Caucasian	Head injuries	ACG, MC, THL
UMB 1649	Control	20	M	22	Hispanic	Multiple injuries	ACG, MC, THL
UMB 1708	Control	8	F	20	African American	Asphyxia, multiple injuries	ACG, MC, THL
UMB 1790	Control	13	M	18	Caucasian	Multiple injuries	ACG
UMB 1793	Control	11	M	19	African American	Drowning	ACG, MC, THL
UMB 1860	Control	8	M	5	Caucasian	Cardiac Arrhythmia	ACG
UMB 4543	Control	28	M	13	Caucasian	Multiple injuries	ACG, MC, THL
UMB 4638	Control	15	F	5	Caucasian	Chest injuries	ACG
UMB 4722	Control	14	M	16	Caucasian	Multiple injuries	ACG, MC, THL
UMB 797	Autism	9	M	13	Caucasian	Drowning	ACG, THL
UMB 1638	Autism	20	F	50	Caucasian	Seizure	ACG, MC, THL
UMB 4231	Autism	8	M	12	African American	Drowning	ACG, MC, THL
UMB 4721	Autism	8	M	16	African American	Drowning	ACG, MC, THL
UMB 4899	Autism	14	M	9	Caucasian	Drowning	ACG, MC, THL
B 5000	Autism	27	M	8.3	NA	NA	ACG, MC, THL
B 6294	Autism	16	M	NA	NA	NA	ACG, MC, THL
B 6640	Autism	29	F	17.83	NA	NA	ACG, MC, THL

a Autism Tissue Program (ATP) identifier.

b Brain regions for which, each sample was available.

M: Male; F: Female, PMI: Postmortem interval, ACG: Anterior cingulate gyrus; MC: Motor cortex; THL: Thalamus; NA: Not available.

### Selection of SNPs

**Figure 3:**
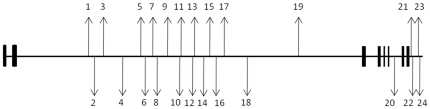
Genomic structure of *LMX1B* gene. Locations of SNPs selected for the association study, based on the HapMap data on Caucasian population, are denoted by arrows. Exons are indicated by boxes.

*LMX1B*, located in 9q33.3 (129,376,748 – 129,463,311), is 86.56kb in size and consists of eight exons. The genomic structure is based on the UCSC (http://www.genome.ucsc.edu) assembly of the human genome. SNPs for the association studies were selected using the information from international HapMap project (http://www.hapmap.org) and National Centre for Biotechnology Information (NCBI dbSNP: http://www.ncbi.nlm.nih.gov/SNP). On the basis of their genomic locations and minor allele frequencies (MAF >0.1), 24 SNPs were selected (Figure 3; Table 1), using the pair-wise tagging option of Haploview.v4.1 (http://www.broad.mit.edu/mpg/haploview).

### Genotyping

Assay-on-demand/Assay-by-design SNP genotyping products (ABI, Foster City, CA, USA) were used to score SNPs, based on the TaqMan assay method [29]. Genotypes were determined in ABI PRISM 7900HT Sequence Detection System (SDS) (Applied Biosystems), and analyzed using SDS v2.0 (ABI).

### Statistical Analysis

PedCheck v1.1 (http://www.watson.hgen.pitt.edu) was used to identify and eliminate all Mendelian inheritance inconsistencies in the trio genotype data. SNPs were tested for Hardy–Weinberg Equilibrium (HWE) using Haploview. SNP associations were examined by transmission disequilibrium test (TDT), using the TDTPHASE option of UNPHASED v2.403 (http://portal.litbio.org); expectation maximization (EM) algorithm was used to resolve uncertain haplotypes, to infer missing genotypes and to provide maximum-likelihood estimation of frequencies.

A linkage disequilibrium (LD) plot was constructed using the D' values. Pair-wise LD values between SNPs were estimated using Haploview. Subsequently, associations of haplotypes (frequency >0.01) belonging to the various haploblocks of *LMX1B* were also examined using Haploview.

### Extraction of RNA from brain tissues

The brain tissues were homogenized by ultrasonication and total RNA was extracted using TRIzol Reagent (Invitrogen, Carlsbad, CA, USA), in accordance with the manufacturer's protocol. The RNA samples were further purified using RNeasy Micro Kit (QIAGEN GmbH, Hilden, Germany), following the manufacturer's instructions. The quantity (absorbance at 260 nm) and quality (ratio of absorbance at 260 nm and 280 nm) of RNA were estimated with a NanoDrop ND-1000 Spectrophotometer (Scrum, Tokyo, Japan).

### Quantitative real-time reverse transcriptase PCR (qRT-PCR)

ImProm-II Reverse Transcription System (Promega, Madison, WI, USA) was used to synthesize first-strand cDNA from the total RNA according to the manufacturer's protocol.

RT-PCR primers for *LMX1B* (NM_001174146.1) (F-cctttgagcaagtaaggataatgaatg, R-gggactgaatttcccagcaa) and endogenous reference *GAPDH* (NM_002046.3) (F-atcagcaatgcctcctgcac, R-tggcatggactgtggtcatg) were designed using primer express v2.0 (Applied Biosystems). SYBR Green qRT-PCR assays were performed using QuantiTect SYBR Green PCR kit (Qiagen). All the reactions were performed in triplicate, in the ABI PRISM 7900HT Sequence Detection System. C_T_ values, which reflect the mRNA expression levels, were determined. *LMX1B* C_T_ of each sample was normalized to the corresponding C_T_ for the internal control by calculating △C_T_ (△C_T_  = Target gene C_T_ – *GAPDH* C_T_) to obtain the relative mRNA expression of the target gene. Quantification of the gene expression was performed by calculating △△C_T_ (△△C_T_  =  △C_T_ of the autistic group - △C_T_ of the control group).The fold change in gene expression between the two groups was determined by calculating 2^−△△C^T.

### Statistical analysis

For the gene expression studies, statistical calculations were performed using PSAW statistics 18.0 software (IBM-SPSS, Tokyo, Japan). The difference in age and postmortem interval between autistic and control groups was examined by t-test. The chi-square test was used to examine the sex distribution; alteration in gene expression between the two groups was analyzed by Mann-Whitney U-test.
